# A Case of Acute Motor Axonal Neuropathy Mimicking Brain Death and Review of the Literature

**DOI:** 10.3389/fneur.2016.00063

**Published:** 2016-04-26

**Authors:** Sandhya Ravikumar, Poysophon Poysophon, Roy Poblete, May Kim-Tenser

**Affiliations:** ^1^Department of Neurology, University of Southern California, Los Angeles, CA, USA

**Keywords:** Guillain–Barré syndrome, brain death, acute motor axonal neuropathy

## Abstract

We describe a case report of fulminant Guillain–Barré syndrome (GBS) mimicking brain death. A previously healthy 60-year-old male was admitted to the neurointensive care unit after developing rapidly progressive weakness and respiratory failure. On presentation, the patient was found to have absent brainstem and spinal cord reflexes resembling that of brain death. Acute motor axonal neuropathy, a subtype of GBS, was diagnosed by cerebrospinal fluid and nerve conduction velocity testing. An electroencephalogram showed that the patient had normal, appropriately reactive brain function. Transcranial Doppler (TCD) ultrasound showed appropriate blood flow to the brain. GBS rarely presents with weakness so severe as to mimic brain death. This article provides a review of similar literature. This case demonstrates the importance of performing a proper brain death examination, which includes evaluation for irreversible cerebral injury, exclusion of any confounding conditions, and performance of tests such as electroencephalography and TCDs when uncertainty exists about the reliability of the clinical exam.

## Background

Guillain–Barré syndrome (GBS) is a term used to describe a group of acute, immune-mediated polyneuropathies that are clinically characterized by rapidly progressive, symmetrical, ascending weakness, and hyporeflexia. The two most common subtypes of GBS are acute inflammatory demyelinating polyneuropathy (AIDP) and acute motor axonal neuropathy (AMAN), with AIDP representing 60–80% of cases (1). The incidence of AMAN is less clear but seems to vary between geographical locations. Its frequency is estimated at 6–7% in the UK and Spain, and 30–65% in Asia, Central America, and South America. It is postulated that geographical variation is due to differences in infectious exposures and genetic susceptibilities. Overall, AMAN is likely underdiagnosed or incorrectly diagnosed as AIDP (1–9).

The degree of muscle weakness in GBS is variable but can be severe with 25% of patients requiring mechanical ventilation from respiratory failure (1, 10–15). In this case report, we describe a patient with fulminant AMAN whose clinical presentation mimicked brain death beginning 7 days after the onset of symptoms, and lasting for 7 days before return of central nervous system function on clinical examination.

## Introduction

A 60-year-old male with diabetes mellitus, hypertension, and chronic obstructive pulmonary disease presented to a local community hospital with worsening shortness of breath. Two days prior, he was discharged from a 2-week hospital stay for community-acquired pneumonia complicated by empyema requiring chest tube placement. There was no history of recent vaccination, diarrheal illness, or recent travel. He was endorsing subjective bilateral lower extremity weakness, back pain, and difficulty urinating on the days leading up to admission.

## Exam, Diagnosis, and Treatment

The initial neurological exam on the day of admission to the community hospital was normal. Later that day, the patient developed ascending right-sided weakness and numbness involving his leg and arm with hyporeflexia noted in the right upper and lower extremities. On hospital day 2, the patient developed ascending left-sided leg and arm weakness and numbness. That day, he developed facial muscle and bulbar weakness that progressed to an inability to speak or eat. Cerebral spinal fluid (CSF) analysis within 48 h of initial presentation revealed an albuminocytologic dissociation, with 0 white blood cells present and a protein level of 103. Laboratory work-up in serum and CSF showed no growth on bacterial cultures and a negative viral and fungal work-up. MRI of the brain, cervical, and lumbar spine was negative for acute changes. On hospital day 3, the patient was started on IVIg for treatment of suspected AIDP. Despite treatment, his condition continued to deteriorate with generalized weakness progressing to respiratory failure requiring intubation. By hospital day 5, cranial nerve function was clinically lost, characterized by absent pupillary responses, corneal responses, vestibulo-ocular reflexes, and gag and cough reflexes. Additionally, the patient had progressed to flaccid quadriplegia and was no longer triggering spontaneous breaths on mechanical ventilation. Based on his clinical exam, there was concern from his providing physician that the patient may have met criteria for brain death, and the family was told that his prognosis was extremely poor and most likely not compatible with life.

**Figure 1:**
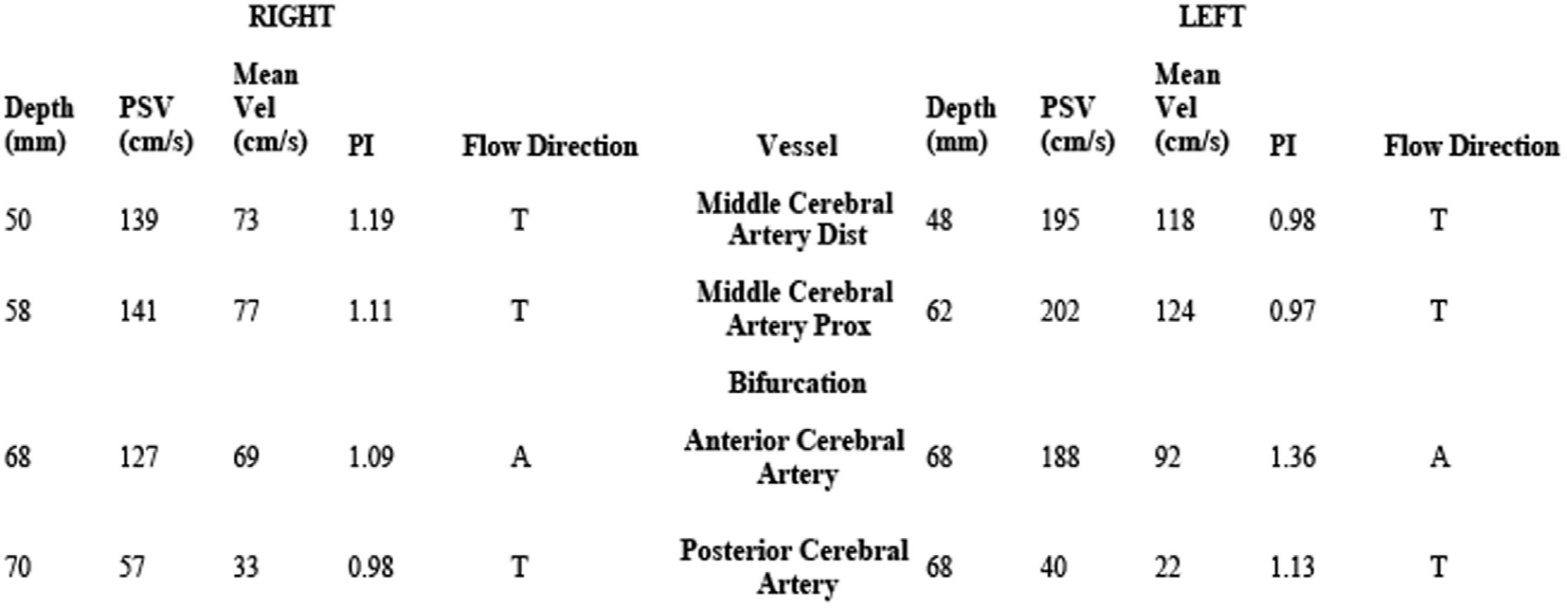
**Transcranial Doppler study shows preserved cerebral blood flow in the middle, anterior, and posterior cerebral arteries**.

**Figure 2:**
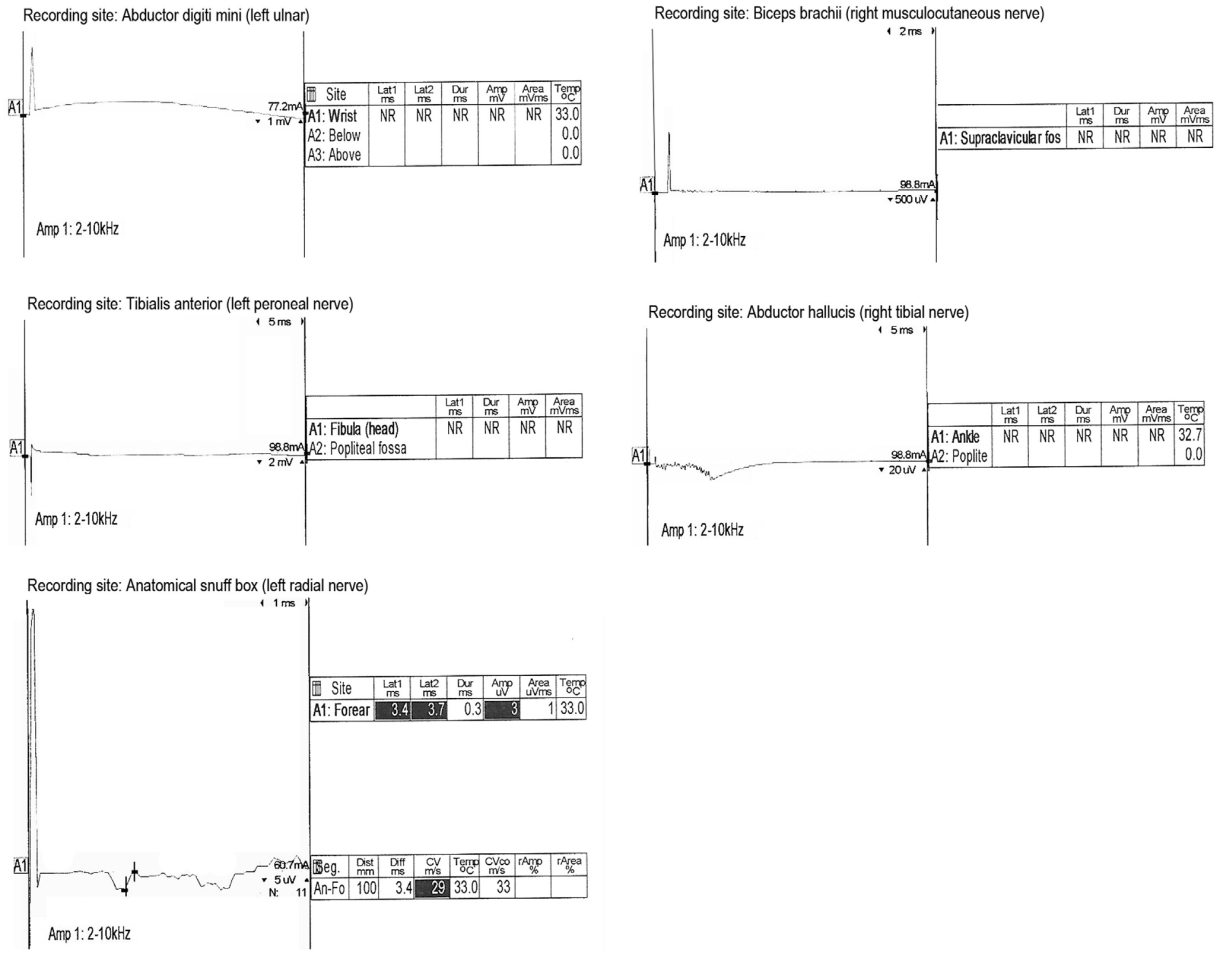
**Nerve conduction results**. Motor responses were absent in bilateral upper and lower extremities. Radial nerve sensory action potential was present but showed delayed latency and reduced amplitude.

On hospital day 6, the patient was transferred to our neuroscience intensive care unit at an academic tertiary care center. Our exam confirmed absence of cranial nerve function, absence of patient-triggered breaths on mechanical ventilation, complete flaccid tetraplegia with lack of response to noxious stimuli, and absence of all deep tendon reflexes. Although the clinical exam mimicked brain death, there was uncertainty about the reliability of the exam because of the lack of history or imaging supporting the presence of catastrophic brain injury. Because of this, a 24-h electroencephalogram (EEG) was completed, which confirmed the presence of cortical brain activity with preserved background rhythms and reactivity. Transcranial Doppler (TCD) ultrasound demonstrated preserved cerebral blood flow (Figure 1). While AIDP remained highest on the differential due to the patient’s pattern of ascending weakness and albuminocytologic dissociation in spinal fluid, other considerations included varicella virus (VZV) neuropathy, West Nile polio-like myelitis, vasculitic neuropathy, and heavy metal poisoning due to the patient’s prior history of working in a plastics factory. VZV PCR in the CSF was undetectable. Serum and CSF West Nile antibody were negative. Vasculitis work-up, including ANA, ANCA, and complement studies, was negative. Heavy metal screen was negative. A lumbar puncture was repeated on hospital day 7 with CSF showing persistent albuminocytologic dissociation (408 RBC; 0 WBC; 339 protein; 121 glucose). Serum anti-GQ1B and anti-GM1 antibodies were negative. A nerve conduction study was performed on hospital day 8, which is presented in Figure 2. Motor action potential responses in bilateral upper and lower extremities were completely absent, while the left sensory radial nerve showed a preserved signal with low amplitude and delayed peak latency, although a full sensory study was difficult to obtain in the ICU setting. The diagnosis based on nerve conduction velocity results was AMAN.

Prior to transfer to our hospital, the patient was treated with empiric IVIg for 3 days for a presumptive diagnosis of AIDP. Because of progression of symptoms despite treatment with IVIg, on hospital day 7, he was switched to plasmapheresis for a 7-day course. The patient began breathing above the set ventilator rate on hospital day 11. On hospital day 14, the patient regained trace, non-purposeful neck movements. On hospital day 19, he began to open and close his mouth spontaneously, and on hospital day 27, he began to follow commands with his mouth and tongue. The patient’s hospital course was complicated by intermittent tachycardia and labile blood pressures suggestive of autonomic instability. He eventually underwent percutaneous tracheostomy and gastrostomy tube placement. Thirty-two days after his initial admission, the patient was discharged to a long-term acute care facility able to move his head, mouth, and tongue to command; however, he remained areflexic and plegic in all four extremities and had complete ophthalmoplegia and non-reactive pupils. Six months after his initial admission, the patient remained in a long-term acute care facility with high ventilator requirements, although he regained proximal arm and leg strength. He was able to sit up in a chair and mouth words to communicate.

## Discussion

This case report describes a patient with fulminant AMAN with progressive loss of appendicular strength and deep tendon reflexes leading to areflexic quadriplegia, respiratory muscle weakness progressing to respiratory failure, and loss of brainstem reflexes. The providers at the community hospital mistakenly thought that the patient’s presentation was consistent with brain death based on (1) absence of spontaneous movements or posturing; (2) absent direct and consensual pupillary light reflexes; (3) absent corneal, oculocephalic, cough, and gag reflexes; and (4) absent oculovestibular reflexes. A formal apnea test likely would have had significant CO2 retention meeting diagnostic threshold due to absent respiratory muscle function requiring full ventilatory support. According to the brain death criteria from the American Academy of Neurology (14), however, before diagnosing brain death, it is a requirement to evaluate for proximate and irreversible injury to the brain and to pursue testing with EEG and TCD ultrasound when uncertainty exists about the reliability of the physical exam or when the apnea test cannot be performed. Based on the lack of evidence for brain injury as well as EEG and TCD studies demonstrating preserved cerebral function, the criteria for brain death were not fulfilled. The diagnosis of AMAN was made by clinical history, albuminocytologic dissociation in CSF, and nerve conduction velocity testing.

The natural course of fulminant AMAN and other GBSs is largely unknown given there are only a few other reports of severe GBS mimicking brain death. Fulminant GBS has been reported in a wide distribution of ages and ethnicities (15–21). In our patient, total absence of motor, respiratory, and brainstem function occurred for a total of 7 days, similar to the cases described by Liik et al. and Friedman et al., although these cases were diagnosed as acute motor and sensory axonal neuropathy (AMSAN) (18, 21). In other cases presented in the literature, electrophysiological studies were also paramount in establishing the diagnosis (15–21). Acute axonal neuropathies, such as AMAN and AMSAN, have been reported to have a rapid progression and a prolonged recovery time compared to acute demyelinating neuropathies (4, 21, 22). The clinical differences may be related to underlying differences in mechanism, as the axolemma and nodes of Ranvier are targeted in AMAN compared to Schwann cells being targeted in AIDP. AMAN is more likely to have positive anti-GM1 and anti-GD1 antibodies compared to AIDP and more likely to be associated with a preceding *Campylobacter* infection, making molecular mimicry a potential etiology (4, 23). While our patient’s rapid and severe presentation is similar to other reports of fulminant acute axonal neuropathy, this is not the classic presentation for AMAN. AMAN typically does not involve cranial nerves and is not typically associated with autonomic lability (4). It remains unknown which factors predispose patients to having more fulminant courses of GBS. Our patient did have the commonly cited complications of GBS, which include autonomic dysfunction presenting as persistent tachycardia, hypertension, gastrointestinal dysfunction, and bladder dysfunction (1, 24). Respiratory insufficiency, pulmonary infection, autonomic dysfunction, and cardiac arrest are the most common causes of death in GBS (1, 25–27). In terms of the treatment of GBS, the only proven effective therapies are IVIg and plasma exchange (1, 3, 28). There are currently no controlled studies suggesting that either IVIg or plasma exchange are superior to one another in treating GBS (1). Furthermore, studies have failed to show that combination therapy with plasma exchange followed by IVIG is more effective than either therapy alone (1, 11, 27). The decision in our patient to start plasmapheresis after 3 days of IVIg was due to clinical deterioration despite IVIg treatment. Motor recovery from severe forms of GBS can be expected to be prolonged, with progressive recovery over a period of months to 3 years being reported (15–19). In contrast, cognitive function and memory may be relatively preserved although some difficulty with recall may be expected (16).

## Concluding Remarks

This case highlights the importance of recognizing fulminant AMAN and other GBSs as a mimicker of clinical brain death and a cause of a “locked-in” state, especially in the absence of catastrophic brain injury. Awareness of the potential clinical manifestations and long-term prognosis of fulminant GBS is crucial for both acute management of these patients and for education and counseling provided to family members and other health-care providers.

## Ethics Statement

The study is a description of a clinical case with a literature review. There were no experimental methods used that would necessitate the approval of an ethics committee. Consent from the patient’s daughter was obtained to allow for publication of this case report. The patient’s daughter was assured that the report would remain anonymous and that no personal identifiers would be used. The patient was not able to give consent due to his clinical condition.

## Author Contributions

SR: compiled references, wrote and edited manuscript, designed figure, and made subsequent draft revisions. PP: wrote initial draft. RP: edited manuscript. MK-T: edited manuscript, senior author.

## Conflict of Interest Statement

The authors declare that the research was conducted in the absence of any commercial or financial relationships that could be construed as a potential conflict of interest.
